# The changing landscape in nephrology education in India

**DOI:** 10.3389/fneph.2023.1110704

**Published:** 2023-02-08

**Authors:** P. S. Vali, Namrata Parikh, Krithika Mohan, Urmila Anandh

**Affiliations:** ^1^ Asian Institute of Nephrology and Urology, Hyderabad, India; ^2^ The Ottawa Hospital, Ottawa, ON, Canada; ^3^ Hosmat Hospitals India, Bangalore, India; ^4^ Department of Nephrology, Amrita Hospitals Faridabad, Delhi, India

**Keywords:** social media, Twitter, digital tools, nephrology education, pitfalls

## Abstract

Digital tools have revolutionized education in nephrology in India. All forms of in-person learning are moving online. Social media have taken over the world, with clinicians learning and promoting multidirectional education methods. E-learning is better equipped to keep up with the rapid pace of new knowledge generation and dissemination. The use of digital multimedia tools to enhance rapid learning is backed by science, viz., dual-coding theory. Digital tools such as Twitter, blogs, podcasts, YouTube, and Nephrology Simulator (NephSIM) have had an impact in facilitating nephrology education among medical professionals and the general public. Digital tools, such as NephMadness, have resulted in the gamification of nephrology learning. Social media usage by the nephrology community in India is growing at a rapid pace. Everyday Cases in Nephrology (#ECNeph), a monthly Twitter-based discussion focused on academically challenging clinical cases, has its origins in India. The Women in Nephrology, India (WIN-India) initiative is very active in facilitating digital education in India and has, in a short space of time, created phenomenal momentum. Furthermore, non-governmental organizations in India, such as the Kidney Warriors Foundation and the Multi Organ Harvesting Aid Network (MOHAN) Foundation, have successfully tapped into social media to educate and aid kidney disease patients. All technologies come with some drawbacks. Despite their acceptance and validation, digital tools have their own pitfalls. These relate to (1) accessibility and connectivity, (2) accuracy of the scientific information, (3) social media noise, and (4) patient privacy. All pitfalls of digital education can be addressed by avoiding excessive social media overload and adopting an appropriate peer-review process. It is advisable to seek written consent from patients whenever patient data are posted online, to avoid privacy issues.

## Introduction

The internet has played a pivotal role in revolutionizing the world and transforming it into a global village: almost 60% of the global population has access to the internet (1). Digital tools have revolutionized education, especially since the emergence of the COVID-19 pandemic. Books are being replaced by e-books, lectures by YouTube videos, and regular in-person discussions by podcasts. Digital tools, especially social media, have taken over the world, with educators developing innovative teaching methods. The target audience of these tools is not only healthcare workers, but also patients and caregivers. With an estimated average daily usage of 147 minutes per person, social media has reinvented the way people communicate and decipher information (2).

Social media are increasingly being used for e-learning, and may be better equipped to keep up with the rapid pace of new knowledge generation and dissemination, as well as the desire for learning at any time and from any location (3). On social media, a network of educators contribute to digital sources by explaining concepts, answering questions, or creating durable content in a variety of formats, whereas others serve as curators, organizing content and directing people to the right answers (4). In a systematic review, it was found that use of social media was associated with improved knowledge, attitudes, and skills (5). The use of social media was also reported to promote collaboration, feedback, and professional development.

This shift to e-learning has also paved the way for the introduction of a diverse set of digital resources, which can be used as educational tools. The medical profession is no exception when it comes to capitalizing on these digital tools to harness mutual learning and educate the public (4).

Several scientific organizations, such as the International Society of Nephrology (ISN) and, locally in India, Women in Nephrology (WIN) and the Indian Society of Organ Transplantation (ISOT), have dedicated social media teams covering their conferences on platforms such as Twitter, Facebook, YouTube, and Instagram, which enables them to reach a wider audience.

## Why digital tools?

The use of a digital multimedia methodology as a learning tool is based on dual-coding theory (6). This theory states that the working memory of a human brain has two parallel channels with regard to information acquisition, processing, and retention: the visual/pictorial channel and the auditory/verbal processing channel. The efficacy of information processing and retention is amplified when both of these channels receive input. Therefore, the purpose of integrating digital tools into medical education is to enhance the efficacy of learning by incorporating both visual and auditory inputs into the conventional medium of teaching.

## Digital tools in nephrology education

The nephrology community took to social media with alacrity, and over the years many platforms have been used to propagate nephrology knowledge. These initiatives have met with considerable success. Presented below is a brief review of the digital tools of greatest importance in the field of nephrology education.

### A: Twitter

Twitter is a digital micro-blogging platform that allows users to post text content in the form of a tweet, which has a maximum character limit of 280 characters. The ability to post text and multimedia content in a tweet is well suited to the delivery of education in small, easily consumed segments. The medical profession quickly adopted Twitter as a medium for educational tools and professional networking. A series of tweets threaded around a core theme constitutes a “tweetorial.” These tweetorials have evolved into interesting educational compilations that provide on-the-spot comprehensive educational content in an “easy-to-grasp” format (4).

Hashtags are also used to maximize Twitter’s potential. A hashtag is used to categorize content in a consistent way. For example, searching for #NephPearls in the Twitter search window will instantly capture and display a wealth of nephrology-related information. In addition to allowing for more focused browsing of educational content, hashtags are used to discuss scientific papers in online journal clubs and to tweet conference-related content. However, in order to reap the greatest benefit from hashtags, one must use popular hashtags. The ability to conduct polls is also a convenient Twitter tool and aids in the gamification of the educational content (7). In addition, @Ask Renal is an innovative Twitter account that provides users with quick answers to questions related to renal sciences. It is a crowdsourced Twitter bot that will automatically propagate any tweets with the hashtag #askrenal (8).

The best part of Twitter is its interface, which allows the expression of content with a limited number of characters and real-time synchronization. These two features aid in the creation of conversations almost in real time, which in turn has made Twitter a dependable tool for hosting real-time scientific conversations and online journal clubs.

### B: Blogs

A blog is a web page with content that is displayed in reverse chronological order and is expected to be more dynamic than a website (9). Every blog has an inbuilt search window that displays all the content related to the search phrase, thereby facilitating the quick retrieval of the content being sought (10).

The *American Journal of Kidney Diseases* (AJKD) blog (https://ajkdblog.org/), maintained under the aegis of the AJKD, is a content-loaded blog. The objective of this blog is to present the journal’s practice-changing content in an engaging format. Another feature of the AJKD blog is the Atlas of Renal Pathology, a rich collection of renal histopathology topics presented in a systematic and lucid format.

Another noteworthy renal blog that deserves special mention is the Renal Fellow Network (RFN). It is a first-of-its-kind, peer-reviewed online forum that is contributed to and maintained by nephrology fellows, and is supervised by faculty advisors (11). It is intended to foster interest in the field of nephrology. The RFN blog has a vast number of bite-sized posts on subjects across clinical nephrology and established a partnership with the American Society of Nephrology (ASN) in 2018 to expand its reach. The Kidney Reports Community is another useful nephrology blog that facilitates the publication of educational content in a visually appealing format (12).

### C: Podcasts

Podcasts are themed collections of digital audio files that are made available for downloading and listening. The ASN podcast hosts interviews with experts, with a focus on advances in nephrology. The National Kidney Foundation’s podcast series “Life as a Nephrologist” focuses on the decision to pursue nephrology as a career and the excitement that comes with it. “Freely Filtered” is a Nephrology Journal Club (NephJC) podcast series and presents a twice-monthly summary of the online journal club. Other interesting nephrology podcasts include “Kidney 360”, “Channel Your Enthusiasm”, “The Nephron Segment”, “NephTalk”, “Throwback Thursday with Dr. Fred Silva”, and “Kidney Essentials”.

### D: YouTube

YouTube is a video-on-demand hosting platform that has had an unprecedented impact in connecting multimedia content with the human race. Not only is it the second most commonly visited website, but it also has a robust user base of more than 2.5 billion monthly users and records more than 1 billion hours of video-watching each day (13).

YouTube is being utilized by educators in nephrology to educate patients, the public, and health professionals, including nephrologists (14). An early such channel was “Nephrology on Demand”, which served as an educational resource for nephrology caregivers and patients. The Glomerular Disease Study & Trial Consortium (GlomCon) YouTube channel is a popular forum that aims to provide a unified platform for all clinicians and pathology researchers interested in glomerular disorders.

It should be noted that, although YouTube is flooded with a wide range of patient education content, very little of it is of high quality. In a study in which 295 peritoneal dialysis videos were evaluated systematically for the quality of their content, only 17% of the videos targeted at patient education were of reliable quality (15).

YouTube has enormous promise as a digital instructional resource, given its accessibility and popularity. However, in a manner similar to the existing popular YouTube Kids app, it is time to develop a subsidiary scholarly section that will permit the display of multidisciplinary academic content in a focused manner. In addition, the time has come for the nephrology community to consider generating patient-centric YouTube videos in multiple local languages.

### E: NephSIM

NephSIM is a digital educational simulator tool (www.nephsim.com) that was launched in 2018. It is essentially a mobile-optimized website that aims to facilitate case-based teaching (16). It is based on a framework of interactive cases and iterative feedback. On this platform, a real-life case will be presented in a systematic pattern in which it is ultimately funneled through a differential diagnosis pathway, following which the final diagnosis and management are discussed. In a survey assessing the impact of NephSIM on academic learning in nephrology, 96% of respondents reported that they enjoyed using NephSIM, and almost all of them intended to continue using this tool in the future (16).

### F: NephMadness—the power of gamification

NephMadness is a popular initiative that uses a gamification strategy to disseminate nephrology information. In this virtual competition, 32 nephrology concepts compete against each other (17). These ideas are chosen to be representative of the most important ideas and scientific papers from the past few years. Structured along the lines of the college basketball playoff model, NephMadness is a single-elimination contest in which concepts compete against one another and participants predict the winning concepts. A distinguished panel of judges selects the winning concepts, and contestants see how their predictions compare. The objective is to facilitate learning about advances in nephrology. The roadmap of NephMadness is structured so that the contestants and their advocates create propaganda on various social media platforms, such as Twitter, the AJKD blog, and podcasts, to increase awareness on their topics of choice. In this way, this virtual gamification strategy amplifies and propagates nephrology education.

The free open-access medical education (FOAMed) community for nephrology has made NephMadness an annual event for the past 10 years. Every year, over a thousand people enter the competition to engage in fun-based learning (18).

### G: Other digital tools

Visual abstracts have revolutionized the way in which work published in medical journals is delivered and disseminated. First introduced by Professor Andrew Ibrahim, visual abstracts aid the viewer in rapid screening of the available scientific articles in a visually appealing format, and can persuade viewers to embark on a deeper analysis of a particular work. Over the past 9 years, all major nephrology journals have adopted visual abstracts as a standard digital supplement to their published content (19, 20).

**Figure 1:**
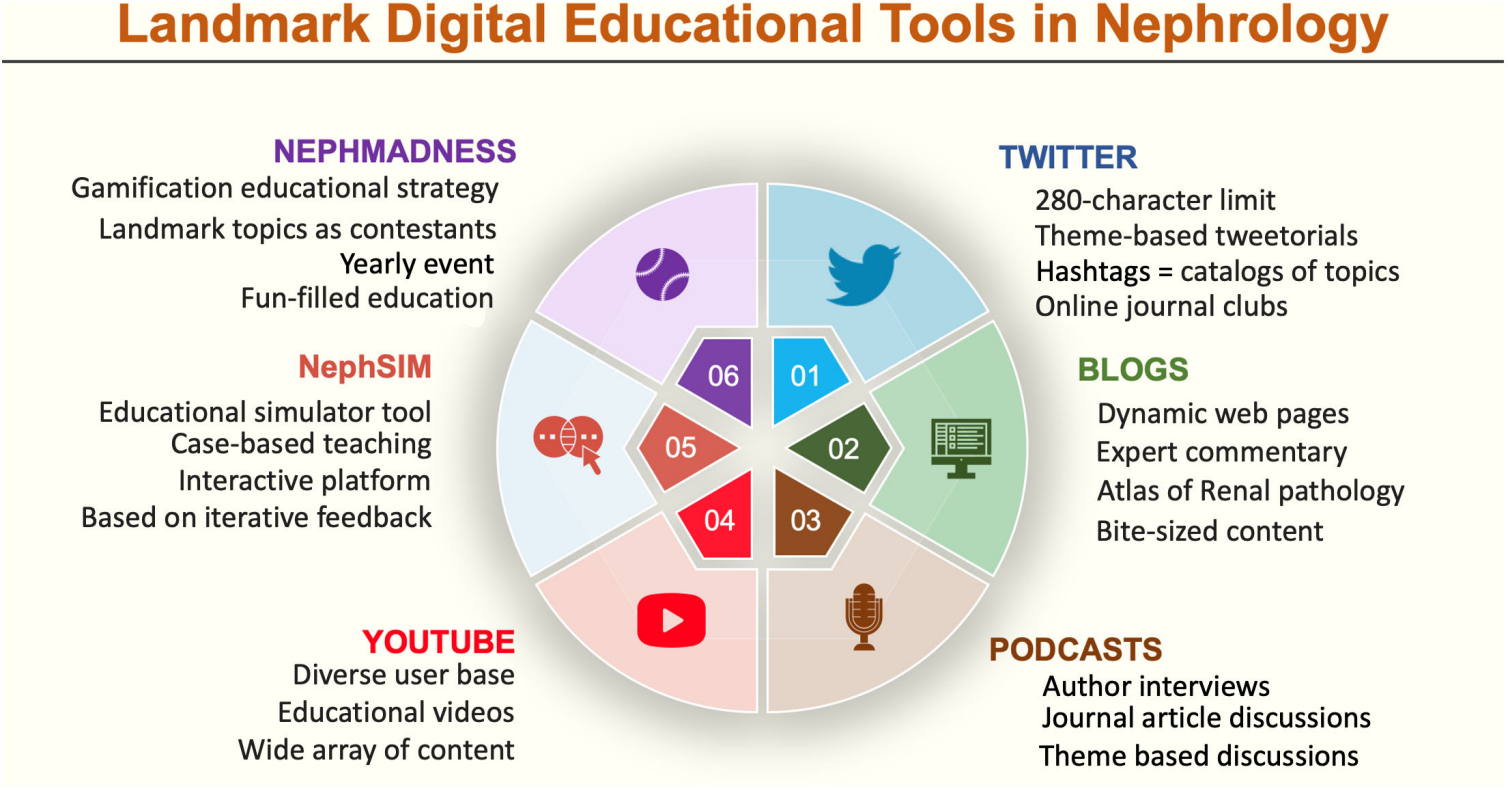
Landmark digital tools in nephrology.

Platforms such as Facebook Live and Zoom allow audiences to interact with the educator and have great potential to facilitate education of target audiences, especially professional peers, patients, and caregivers, in a flexible way (21). Questions are answered, concepts are explained, and expert feedback is provided to learners, thus providing a digital mentorship (4). Instagram and LinkedIn allow two-way conversations, although not in real time. The major digital tools in nephrology education are depicted in **Figure 1**.

## Digital education of patients and caregivers

Over 850 million people worldwide are affected by chronic kidney disease (CKD) (22). Digital education has been used to create awareness of CKD in the general population. Freely available applications (apps) such as the app by the Renal Health Project aim to help patients undergoing dialysis or a renal transplant better understand their disease, organize their treatment, and control their tests, diet, and all aspects of their therapy (23). The Renal Health Project also provides regular health education for patients through social networks such as YouTube and Instagram. This helps them learn more about all aspects of their kidney disease, including risk factors, causes, prevention, treatment, dialysis, and transplantation. Through these projects, patient self-care and management have been shown to improve. Several phone apps, such as “Care after Kidney Transplant”, “Kidney Diet”, and “Find My Dialysis”, are available to help disseminate information to patients (24).

Several organizations, such as the Kidney Warriors Foundation, which is an India-based network of kidney patients, caregivers, healthcare professionals, and social workers, utilize social media widely to advocate for policies that improve access to quality healthcare for patients with kidney disease (25).

## Nephrology and social media—the Indian experience

In January 2022, the number of internet users in India (that is, the number of people with access to digital information) was estimated at 658 million, about 47% of the Indian population. Various social media platforms, such as Facebook, Twitter, Instagram, and LinkedIn, enjoy tremendous popularity in India. In January 2022, of these 658 million citizens with internet connectivity, 467 million were actively participating in social media (26). These statistics demonstrate the immense potential of social media in India. If this is harnessed effectively, it could have far-reaching consequences.

Healthcare professionals in general, and nephrologists in particular, have come to realize this potential and, in recent years, have brought about a minor revolution in our country. Social media have eliminated geographic barriers, and Indian nephrologists have taken advantage of this. For example, the International Society of Nephrology has a dedicated social media team that includes many nephrologists of Indian origin. They have worked together on several occasions and published their experiences (27–29).

The use of online digital tools in nephrology education in India began with the Twitter-based chat “Everyday Cases in Nephrology” (#ECNeph), where academically challenging real- world cases are discussed, with strict measures to ensure patients’ privacy. This initiative quickly gained popularity and won the NephJC Kidneys award for Nathan Hellman Social Media Project of the Year 2017 (30). Now in its fifth year since its conception, #ECNeph continues to draw audiences from several countries for its monthly discussions (31).

“Last Month in Nephrology” is a similar brainchild of Indian nephrologists. It takes the form of a newsletter that provides commentary on recently published evidence. The aim is to change the way nephrologists approach patient care, armed with recent advances in their subspecialty (32).

Indian nephrologists were among the first to use the services of dedicated social media teams to report on their conferences. In 2017, the Association of Vascular Access and inTerventionAl Renal Physicians (AVATAR) conference attracted extensive social media coverage (33). Since then, numerous conferences have achieved excellent coverage in the form of live tweets and livestreams (34, 35).

The Women in Nephrology, India (WIN-India) initiative, launched in 2021, aims to offer mentorship to many aspiring early-career nephrologists. Since its launch, WIN-India has successfully organized several impactful teaching activities. Recently, it held its first international conference Women IN Nephrology-India Conference (WIN-ICON) as a hybrid program, where female nephrologists in the early stages of their careers were given the opportunity to share the stage with speakers of international renown. This is a good example of how social media can bring together mentors and mentees from different corners of the world.

In India, social media tools have also been successfully used for advocacy, especially in the area of organ donation. The Multi Organ Harvesting Aid Network (MOHAN) Foundation in India utilizes Facebook as a channel to drive organ donation. Many non-governmental organizations use social media to create awareness of organ donation. All these organizations practice a certain amount of tact and discretion in their activities, to avoid the spread of misinformation. In 2021, Basu et al. created “best practice” recommendations for social media use concerning organ donation (36). These guidelines can serve as a foundation for the use of social media for advocacy of other initiatives as well.

## Pitfalls of digital education

It is also important to realize the tremendous impact of social media and the implications of social media misuse. The message that can be disseminated to 50 million people via television over 13 years can be disseminated through social media to the same number of people in 3 months. (37) This makes it very important to maintain a strong sense of responsibility and restraint while utilizing these apparently “fun” tools, and to be aware of the pitfalls of this social media. These pitfalls are summarized in **Table 1**.

**Table 1:** Pitfalls of social media use.

Pitfalls	Comments
Accessibility, connectivity, and adaptability	40% of the world still lacks basic internet facilities (e.g., countries with lower socioeconomic status and remote places). Digital tools are popular among the younger population, but elderly people may still hesitate to be a part of this revolution. There are many who are not willing to do away with traditional educational training.
Accuracy of scientific information	Traditionally, data undergo peer review by experts in the field before publication. With digital education, this is not the case; the accuracy of the information can be questioned. Bias may occur in the form of opinions and inaccuracies. Exceptions exist, such as NephJC and NephMadness, which are internally peer reviewed. Large numbers of “fake messages” are circulated on social media.
Social media noise	Constant buzzing and pinging of posts, notifications, and messages leads to anxiety and exhaustion. This can cause time to pass without awareness, resulting in anxiety and exhaustion (31). Social media use can result in a sense of unhealthy competition and can increase peer pressure as users are exposed to a high volume of knowledge.
Patient privacy	Infringement of patient privacy is a major concern in the evolving digital world. Often, patient information is shared in order to disseminate knowledge to learners (often without consent).

In response to this issue, the Nephrology Social Media Collective (NSMC) provides a dedicated year-long internship promoting the effective use of social media, which alerts its students to the pitfalls of social media (38, 39). Some of the leading names in the Indian social media scene have participated in this program and now hold important positions in the faculty of the NSMC.

## Future directions

While Indian nephrologists have been quick to engage with social media, there still appears to be a significant gap between its potential and the extent to which it has been harnessed. Possible reasons include a lack of awareness and interest. The near absence of formal peer review for the majority of posts is also a concern. Clearly, all these lacunae will have to be addressed before we can move forward as “digital” nephrologists. Furthermore, digital tools have been proven to increase treatment adherence in a few non-renal chronic disorders (40). Therefore, it would be prudent to develop such digital tools and algorithms aimed at enhancing treatment adherence among patients with chronic kidney disease.

## Author contributions

All authors listed have made a substantial, direct, and intellectual contribution to the work, and approved it for publication.
